# BRM Complex in Arabidopsis Adopts ncBAF-like Composition and Requires BRD Subunits for Assembly and Stability

**DOI:** 10.3390/ijms24043917

**Published:** 2023-02-15

**Authors:** Paulina Stachula, Katarzyna Kapela, Ewelina Malecka, Kamila Jaronczyk, Jacek Patryn, Nina Siwirykow, Maria Bucholc, Malgorzata Marczak, Maciej Kotlinski, Rafal Archacki

**Affiliations:** 1 Laboratory of Systems Biology, Faculty of Biology, University of Warsaw, Pawinskiego 5A, 02-106 Warsaw, Poland; 2 Institute of Biochemistry and Biophysics PAS, Pawinskiego 5A, 02-106 Warsaw, Poland

**Keywords:** chromatin, Arabidopsis, protein complex, chromatin remodeling, SWI/SNF, mass spectrometry, BRM, BRD, BDH

## Abstract

ATP-dependent SWI/SNF chromatin remodelling complexes are conserved multi-subunit assemblies that control genome activity. Functions of SWI/SNF complexes in plant development and growth have been well established, but the architecture of particular assemblies is unclear. In this study, we elucidate the organization of Arabidopsis SWI/SNF complexes formed around a BRM catalytic subunit, and define the requirement of bromodomain-containing proteins BRD1/2/13 for the formation and stability of the entire complex. Using affinity purification followed by mass spectrometry, we identify a set of BRM-associated subunits and demonstrate that the BRM complexes strongly resemble mammalian non-canonical BAF complexes. Furthermore, we identify BDH1 and 2 proteins as components of the BRM complex and, using mutant analyses, show that BDH1/2 are important for vegetative and generative development, as well as hormonal responses. We further show that BRD1/2/13 represent unique subunits of the BRM complexes, and their depletion severely affects the integrity of the complex, resulting in the formation of residual assemblies. Finally, analyses of BRM complexes after proteasome inhibition revealed the existence of a module consisting of the ATPase, ARP, and BDH proteins, assembled with other subunits in a BRD-dependent manner. Together, our results suggest modular organization of plant SWI/SNF complexes and provide a biochemical explanation for mutant phenotypes.

## 1. Introduction

Changes in nucleosome structure and positioning can be achieved by ATP-dependent chromatin remodeling. This activity is mediated by conserved multi-subunit complexes assembled around central SNF2-type ATPase that uses the energy derived from ATP hydrolysis to change interactions between histone octamers and the DNA [1]. SNF2 proteins can be organized into subfamilies distinguished by structural properties of the catalytic domain and the unique composition of other domains [2,3]. The four main subfamilies are SWI/SNF, ISWI, INO80/SWR, and CHD. Typically, remodeling ATPase is associated with other subunits that are believed to participate in the assembly of complexes, regulation of ATPase activity, and recruitment to target loci [1].

SWI/SNF complexes are the most thoroughly studied remodelers in yeast, plants, and animals. Arabidopsis has homologs for all the main SWI/SNF members, including conserved subunits in all eukaryotes (ATPase, SWI3, SWP73, SNF5, actin-related proteins). Most of them are encoded by gene families; there are four ATPases: SPLAYED (SYD), BRAHMA (BRM), CHR12, and CHR23 (also called MINU1/2) [4,5,6]; four SWI3 subunits (SWI3A, B, C, and D) [7], and two SWP73 subunits (SWP73A and B) [8,9]. Most likely, subunit paralogs enable the combinatorial assembly of different complexes that perform specialised functions. Consistent with the presence of multiple SWI/SNF variants, diverse sets of SWI/SNF components and their paralogs were isolated in IP/MS experiments using tagged versions of SWI/SNF subunits or SWI/SNF-interacting proteins as bait [10,11,12,13,14]. Analyses of Arabidopsis mutants in different subunits indicate that SWI/SNF complexes are essential for transcriptional control of key developmental processes, including seed maturation and embryogenesis, cotyledon separation, cell division during leaf development, maintenance of root stem cell niche, floral patterning, and flowering initiation, as well as abiotic stress responses [15,16]. They have also been shown to participate in the regulation of hormonal pathways, including those of gibberellins (GAs) and abscisic acid (ABA) [17,18].

Among the four SWI/SNF catalytic subunits in Arabidopsis, BRM is considered to be the closest homolog of yeast and animal ATPases, as it contains an acetylated histone-binding motif called bromodomain [3]. Recently, novel members of BRM-associated SWI/SNF complexes (BRM complexes hereafter) have been characterized, including two BRIP proteins (BRIP1/2) ([14] and three bromodomain-containing subunits BRD1/2/13 which are homologs of mammalian GLTSCR1/1L and BRD7/9, respectively [11,19]. BRIP and BRD were found to interact with several core SWI/SNF components and be necessary for the proper regulatory functions of SWI/SNF. Interestingly, *brip1/2* mutants showed decreased protein levels of BRM ATPase and other subunits, suggesting the involvement of BRIP1/2 in the maintenance of the SWI/SNF complex [14]. The triple *brd1/2/13* (*brdx3*) mutation also results in the downregulation of BRM protein levels [11,19], but it is currently unknown whether and how BRD subunits contribute to the formation and/or stabilization of the whole complex. The organisation and abundance of individual BRM assemblies also remain elusive.

To elucidate the organisation of BRM-containing SWI/SNF complexes in Arabidopsis and the contribution of BRD subunits to the integrity of the complex, we combined immunoprecipitation followed by mass spectrometry (IP/MS) experiments with mutant analyses. A highly reproducible isolation of BRM complexes shows that from the spectrum of combinatorial possibilities, BRM associates only with a limited number of subunits to form complexes that strongly resemble the subtype of mammalian SWI/SNF known as non-canonical BAF. Furthermore, we identified the BDH1 and BDH2 proteins, homologs of mammalian BCL7 subunits, as components of BRM and other SWI/SNF complexes. Analyses of single and double mutants indicate that BDH1 and 2 act redundantly to control vegetative development and flowering, as well as responses to GA and ABA. We further show that the bromodomain-containing BRD1/2/13 proteins represent unique components of the BRM complexes and are crucial for the integrity and stability of the complex. Finally, IP/MS experiments in *brd* mutant backgrounds reveal the existence of a catalytic module composed of BRM ATPase, ARP4 and 7, and BDH1/2 proteins, which is assembled with the rest of the complex in a BRD-dependent manner. Although BRD1/2/13 subunits act redundantly, the contribution of each BRD paralog in the formation of BRM complexes is not equal. Together, our studies provide insight into the organization of BRM complexes in plants, the evolutionary relations with their animal counterparts, and the biochemical explanation of reported mutant phenotypes.

## 2. Results

### 2.1. Isolation of the SWI/SNF Complexes Associated with BRM Identifies BCL7-like Proteins

**Figure 1:**
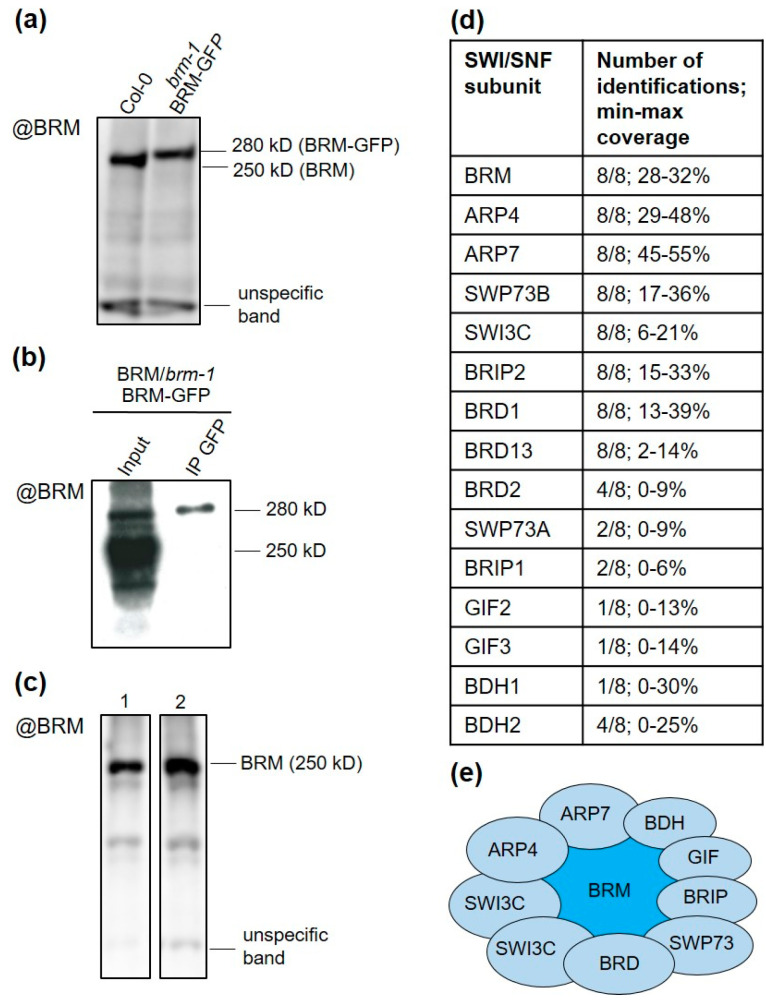
Isolation of BRM complexes from Arabidopsis seedlings: (**a**) Western blot analysis of native BRM and BRM-GFP protein levels in 14-day-old WT and *brm-1/BRM-GFP* plants. The BRM null mutant *brm-1* was used as a negative control. (**b**) Immunoblot showing the precipitation of BRM-GFP from the whole-cell extracts of BRM/*brm-1 BRM-GFP^+/−^* heterozygous plants. Signals corresponding to native BRM (250 kD) and BRM-GFP (280 kD) are visible in the input sample. (**c**) Immunoblot showing native BRM levels in nuclei-enriched (1) and whole cell extracts (2) from 14-day-old WT plants grown in soil or MS medium, respectively. (**d**) SWI/SNF subunits co-purified with BRM-GFP in IP/MS experiments: the number of identifications and min–max coverage for each subunit are shown. (**e**) Composition of the SWI/SNF complex formed by BRM ATPase.

To obtain the complete composition of native SWI/SNF complexes associated with BRM, we first performed a series of immunoprecipitation mass spectrometry experiments (IP/MS) using the Arabidopsis line expressing C-terminal fusion of BRM-GFP under its native promoter in the background of the *brm-1* knockout mutant [11]. As homozygous *brm-1/BRM-GFP* plants are deprived of endogenous BRM, we selected the line in which the level of BRM-GFP protein was similar to that of native BRM in wild-type (WT) plants, as assessed by Western blotting (Figure 1a). BRM-GFP was successfully immunoprecipitated from whole cell extracts, even when present in low amounts in heterozygous plants BRM/*brm-1 BRM-GFP^+/−^* (Figure 1b). To achieve efficient purification of BRM-associated complexes, we used 14-day-old plants grown in vitro (in liquid ½ MS medium), as the level of BRM protein is high in whole cell extracts under these conditions, without the need for nuclei enrichment (Figure 1c). To minimize the degradation of BRM and other subunits of the complex, we applied a single-step rapid protein extraction and a short incubation time with anti-GFP resin (Figure S1a). IPs were performed without the crosslinking step to reduce unspecific protein binding. We confirmed that the known SWI/SNF subunits were co-purified with BRM-GFP using these conditions (Figure S1b).

To analyze the composition of BRM complexes, proteins present in IPs were subjected to on-bead digestion and identified using LC-MS analysis. Nonspecific proteins (contaminants) were extracted by comparing the data from each purification with a mock control (Arabidopsis line expressing only GFP). Analysis of IP/MS results showed that BRM-GFP was co-purified with a distinct set of subunits, namely: two actin-related proteins—ARP4 and ARP7, one SWI3-type protein—SWI3C, one SWP73-type protein—SWP73B, one BRIP—BRIP2, and bromodomain containing proteins BRD1 and BRD13 (Figure 1d; Supplementary Data S1), confirming previous results [11,14]. The identifications were highly reproducible for this set of subunits, as they were found in eight of eight IP experiments. Three other known subunit paralogs, BRD2, SWP73A, and BRIP1, were found with considerably lower identification frequency (in four, two, and two experiments, respectively) and lower protein coverage (Figure 1d), suggesting that BRD2, SWP73A, and BRIP1 are present in relatively small fractions of complexes isolated from the samples. We did not reproducibly identify chromatin proteins known to interact with SWI/SNF complexes, such as REF6 [12], nor SWI/SNF—interacting transcription factors. This indicates that the IP conditions we used favored strong intracomplex interactions and did not preserve weak or transient interactions between SWI/SNF subunits and other proteins. Furthermore, the AN3 (GIF1) protein that was previously shown to associate with SWI/SNF complex subunits including BRM [13] was not identified. However, we could find its GIF2 and GIF3 paralogues in two experiments (Figure 1d). The low identification frequency of GIF proteins may be due to their small size resulting in a low number of peptides that can be analyzed by mass spectrometry, to the weaker nature of GIF interactions with the SWI/SNF complex (compared with other subunits), or to both. Importantly, and in agreement with previous results [13], we could identify SWI/SNF components, including BRM and SYD ATPases, in reciprocal IP/MS experiments performed using the GFP-AN3 overexpressing line (Supplementary Data S2).

Apart from known SWI/SNF subunits, no other proteins were identified in more than two IPs using BRM-GFP, with the exception of a protein encoded by the AT5G55210 gene, which was detected in four experiments (Figure 1d, Supplementary Data S1). AT5G55210 and its close homologue AT4G22320 have recently been proposed to represent orthologs of BCL7 subunits of mammalian SWI/SNF complexes and named BCL domain homolog 1 (BDH1) and BDH2, respectively [20]. Consequently, our comparative sequence analysis showed considerable similarities between BDH1/2 and mammalian BCL7A/B subunits (Figure S2A), and we confirmed nuclear localization of both proteins (Figure S2b). Together, these results suggest that BDH1/2 proteins represent components of BRM complexes (Figure 1e).

### 2.2. Bdh Mutants Display Similar Phenotypic Traits to Other Mutants in SWI/SNF Subunits

**Figure 2:**
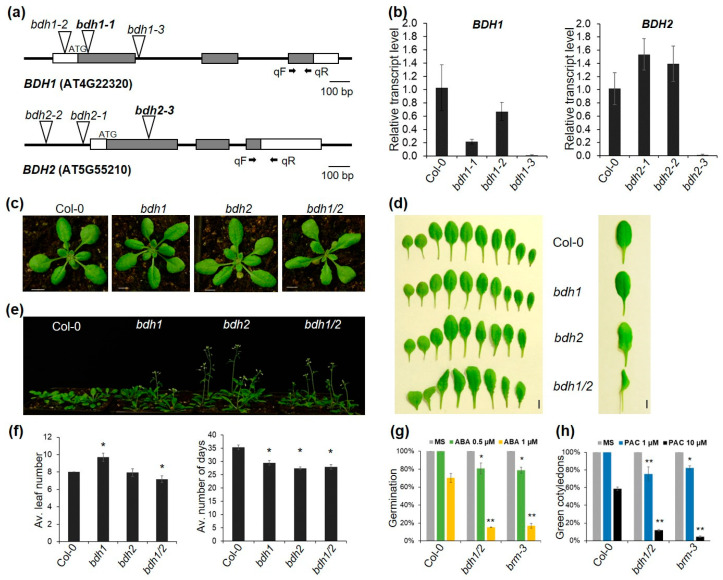
Characterization of *bdh* mutants: (**a**) positions of T-DNA insertions in the *bdh1* and *bdh2* mutant alleles; gray boxes, coding regions; white boxes, UTRs; black lines, introns; black arrows, primers used in RT-qPCR; the mutant alleles used to generate double *bdh* mutants are in bold; (**b**) relative expression levels of *BDH1* and *BDH2* measured by RT-qPCR in 21-d old WT (Col-0) and mutant plants; the PP2A housekeeping gene was used as normalization control; transcript levels in Col-0 are set to 1; error bars indicate the SD of three independent biological replicates; (**c**) rosette phenotypes of 22-day-old Col-0 and mutant plants; bar, 10 mm; (**d**) leaf series of 28-day-old wild-type and *bdh* mutants (left) and rosette leaves from 42-day-old *bdh1/2* mutants showing a strong curling phenotype compared with wild-type and single mutants (right); bar, 10 mm; (**e**) wild-type and *bdh* mutant plants flowering under long-day conditions; the picture was taken on the 32nd day of growth; (**f**) number of leaves at flowering and flowering time under long-day conditions; values are mean ± SD; the asterisks indicate significant differences between the WT and mutant lines (Student’s *t*-test, *p* < 0.01); (**g**) percentage of germinated embryos that develop green cotyledons in the presence of 0.5 or 1 µM ABA; values are mean ± SD; asterisks indicate significant differences from the WT (χ^2^ test, *n* = 50, * *p* < 0.01, ** *p* < 0.001); (**h**) germination assay of wild-type (Col-0) and *bdh* mutants in the presence of different concentrations of PAC; radicle emergence after 7 days was scored as germination; values are mean ± SD; asterisks indicate significant differences from the WT (χ^2^ test, *n =* 50, * *p* < 0.01, ** *p* < 0.001).

As functional data for BDH1/2 were not available, we identified and analyzed T-DNA insertion mutant lines for the two genes. Mutant alleles *bdh1-1*, *bdh1-2,* and *bdh1-3* (SALK_152173, SALK_053046, and SALK_046333, respectively) carried T-DNA insertion in the first exon, promoter, and first intron of *BDH1*, respectively. In alleles *bdh2-1*, *bdh2-2,* and *bdh2-3* (SALK_060883, SALK_042826, and SALK_029285, respectively), T-DNA insertions were located in the promoter and first exon (Figure 2a). Quantitative RT-PCR (RT-qPCR) amplification of the *BDH1* and *BDH2* transcripts located downstream of insertions indicated that the *BDH1* transcript was approximately five-fold lower in the homozygous *bdh1-2* mutant compared with the wild type and undetectable in the homozygous *bdh1-3* mutant (Figure 2b). The transcript level of *BDH2* was slightly upregulated in the homozygous *bdh2-1* and *bdh2-2* mutants compared with the wild type, and undetectable in the homozygous *bdh2-3* mutant (Figure 2b). Furthermore, we were unable to detect full-length transcripts of the mutated genes in the lines *bdh1-2*, *bdh1-3*, and *bdh2-3* (Figure S3). We conclude that *bdh1-2* and *bdh1-3*, as well as *bdh2-3*, are likely null mutant alleles.

The soil-grown null mutants *bdh1* and *bdh2* did not show visible phenotypes at the seedling and rosette stage (Figure 2c). However, at the later stage of growth, the *bdh2* mutants could be distinguished as they produced individual leaves with slightly enhanced curvature (Figure 2d). Furthermore, both single *bdh* mutants flowered earlier than WT plants (Figure 2e). We also noticed alterations in the development of floral organs in these mutants consisting in changes in the number of stamens (Table S1). To test whether *BDH* genes have overlapping functions in development, we crossed *bdh1-1* and *bdh2-3* mutants. The *bdh1/2* double mutant differed considerably from wild type and single mutants by displaying enhanced leaf curvature and downward curling (Figure 2c), which became more pronounced as plant growth progressed (Figure 2d). Furthermore, the double mutants showed earlier flowering (Figure 2e). Under long-day conditions (16 h light/8 h dark), *bdh1*/2 plants bolted about 5 days earlier with about 7 rosette leaves, compared with the wild type, which bolted after producing an average of 8 leaves, the differences being statistically significant (Figure 2f). Examination of flowers revealed that, compared with single mutants, the double *bdh1/2* mutation increased the frequency of flowers with a reduced number of stamens (Table S1). However, we did not observe any major alterations in the other floral organs, and the mutants remained fully fertile. It is important to note that the leaf phenotype and changes in the number of floral organs observed in *bdh1/2* mutants are characteristic of several previously characterized Arabidopsis mutants in different SWI/SNF subunits [4,7,11,19]. Next, we examined the responses of *bdh1/2* mutants to abscisic acid (ABA) and paclobutrazol (PAC, an inhibitor of gibberellin biosynthesis), as mutants in genes encoding SWI/SNF subunits, including BRM, SWI3C, and BRD, were shown to be hypersensitive to ABA and PAC treatments [11,21,22,23]. Compared with the wild type, *bdh1/2* double mutant plants were hypersensitive to ABA, as indicated by inhibition of cotyledon expansion and greening (Figure 2g and Figure S4). Similarly, PAC treatment of *bdh1/2* mutants inhibited seed germination more strongly than in the wild type (Figure 2h). The suppressive effects of ABA and PAC on growth were similar in *bdh1/2* plants to those observed in the *brm-3* mutant that was used here as a control (Figure 2g,h). These results indicate that, similarly to the main SWI/SNF core subunits, BDH1 and 2 regulate the ABA and GA responses. Together, the phenotypic characteristics of the *bdh* mutants are consistent with BDH1 and 2, representing SWI/SNF subunit paralogs with primarily redundant functions in controlling Arabidopsis development.

### 2.3. BRM-Associated SWI/SNF Complex Is Homologous to Mammalian ncBAF

**Figure 3:**
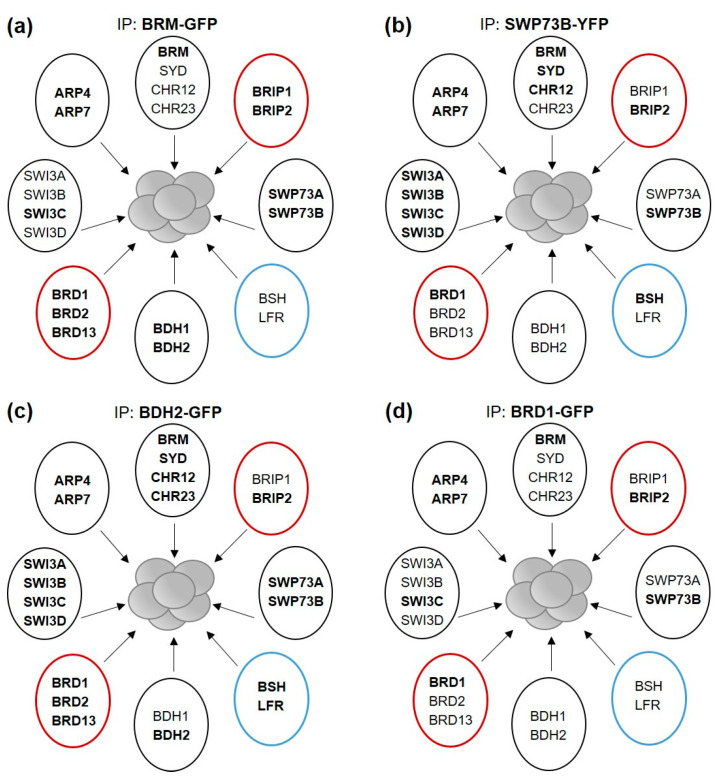
BRM forms ncBAF-like complexes. Subunits of Arabidopsis SWI/SNF complexes identified in IP/MS experiments using BRM-GFP (**a**), SWP73B-YFP (**b**), BDH2-GFP (**c**), or BRD1-GFP (**d**) as bait. Proteins co-purified with each of the baits are shown in bold. Subunit paralogs are grouped by circles, with the exception of LFR and BSH, which do not represent homologs. Red and blue circles represent counterparts of mammalian proteins specific to ncBAF and BAF/PBAF complexes, respectively. Only proteins for which functional data exist are included.

We noticed that a number of known SWI/SNF members, including SWI3A/B/D [7], BSH (an SNF5 homolog) [24], and LFR (a homolog of ARID1/2 subunits) [20,25], were not detected in the IP/MS experiments using BRM-GFP (Figure 3a, Table S2). We reasoned that the subunits frequently identified in BRM-GFP IPs represent the most abundant BRM complex, while the absent subunits are part of separate SWI/SNF complexes containing SYD, CHR12, or CHR23, but not BRM ATPase. Alternatively, a set of subunits was not present in the samples or could not be detected under our experimental conditions. To distinguish between these possibilities, we performed IP/MS experiments using SWP73B-YFP-HA line [8], as SWP73B was previously shown to co-immunoprecipitate many SWI/SNF subunits and paralogs from Arabidopsis cell cultures [13]. Consistent with the presence of SWP73B in different SWI/SNF complexes, multiple known SWI/SNF subunits were identified in SWP73B affinity purifications, including BSH, all SWI3 paralogs (SWI3A/B/C/D), and three ATPases (BRM, SYD, CHR12) (Figure 3b, Table S2, Supplementary Data S3). These data demonstrate that SWI/SNF complexes with different content can be reliably isolated using our experimental setup. Therefore, we concluded that BRM-GFP purifications captured the prevalent BRM complex with a specific native composition. Importantly, the characteristics of this complex strikingly resemble the non-canonical class (ncBAF) of mammalian SWI/SNF complexes [26,27], confirming recent predictions that ncBAF-like assemblies are also present in plants [11,14,20]. The BRM complex in Arabidopsis similarly to mammalian ncBAF does not contain homologous subunits of BAF47 and ARID (BSH and LFR, respectively) but incorporates homologs of GLTSCR (BRIP in Arabidopsis) and bromodomain-containing BRD proteins (BRD1/2/13), as well as a single variant of the SWI3 subunit (SWI3C in Arabidopsis), most likely forming a homodimer (Figure 1e and Figure 3a).

To further investigate the identity of the BRM complex, we performed additional affinity purifications using as bait BDH2-GFP and BRD1-GFP, for which we generated stable homozygous lines in knockout mutant backgrounds (pBDH2:BDH2-GFP/*bdh2* and 35S:BRD1-GFP/*brd1*, respectively). As mammalian homologs of BDH2 were found in all types of SWI/SNF complexes [27], we hypothesized that BDH2 could also be a component of different SWI/SNF complexes in Arabidopsis. On the other hand, BRD1 together with BRD2 and 13 could represent specific subunits of BRM-associated complexes, since the *brm-1 brdx3* quadruple mutant displays the same phenotype as single *brm-1* [11,19]. According to their different features, BDH2, similarly to SWP73B, would be expected to capture a broad spectrum, while BRD1 would capture a narrow set of SWI/SNF subunits. Mass spectrometry analysis confirmed this prediction, as multiple SWI/SNF components were identified in BDH2 affinity purifications (Figure 3c, Table S2, Supplementary Data S4). In contrast, the BRD1 captured complexes containing only BRM ATPase, and, moreover, all other identified subunits overlapped with those obtained in the BRM-GFP purifications (Figure 3d, Table S2; Supplementary Data S5). The combined results demonstrate that SWP73B and BDH2 are incorporated into different SWI/SNF complexes, while BRD1 appears to be a signature subunit of the ncBAF-like complex formed by BRM.

### 2.4. Mutations in BRD Genes Differentially Affect BRM Protein Levels

**Figure 4:**
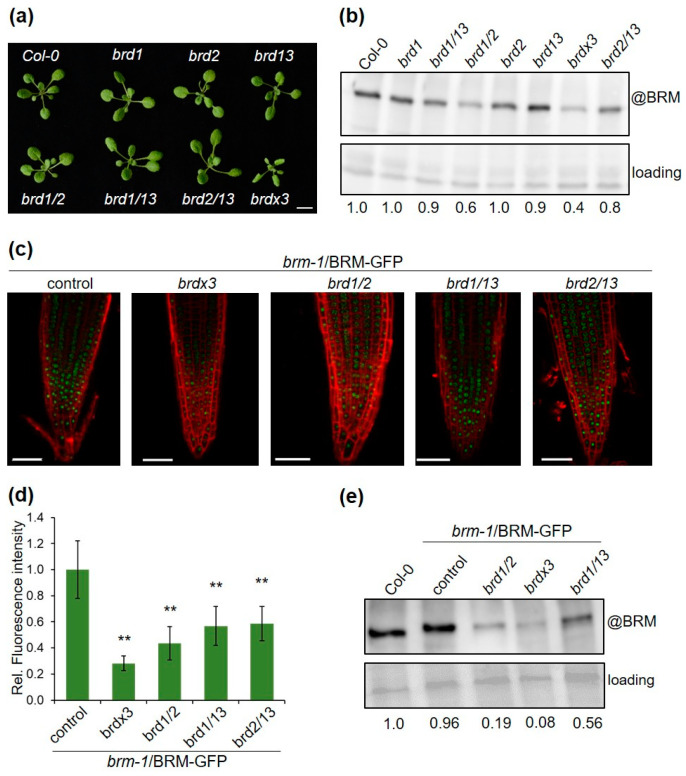
BRD mutations affect BRM protein levels: (**a**) 18-day-old Col-0 and *brd* mutant plants; Bar, 10 mm; (**b**) Western blot analysis of the native BRM protein in 10-day-old Col-0 and mutant plants; signal intensities normalized to the loading control are shown by numbers below the figure; (**c**) images of the root tips of lines expressing BRM-GFP in different backgrounds of *brd* mutants; propidium iodide was used to counterstain the cell walls; Bar = 50 µm; (**d**) relative fluorescence intensity of BRM-GFP in roots of BRM-GFP-expressing lines; values are mean ± SD of 14 measurements from each line; asterisks indicate the significant difference between the *brm-1*/BRM-GFP control line and the *brd* mutant backgrounds (Student’s *t* test, *p* < 0.01); (**e**) immunoblot analysis of the BRM-GFP protein in different genetic backgrounds using anti-BRM antibody. The native BRM is visible in Col-0; signal intensities normalized to the loading control are shown below.

It has recently been shown that the BRD1, 2, and 13 subunits of the BRM complex have redundant functions in controlling gene expression and Arabidopsis development [11,19]. This is reflected in the *brd* mutant phenotypes, since single and double mutants show only minor morphological changes compared with the wild type, while the *brd/1/2/13* phenotype (*brdx3*) is much stronger (Figure 4a). The amount of native BRM protein has also been shown to decrease significantly in the *brdx3* mutant compared with wild type [11,19]. To test which of the BRDs are necessary for maintaining proper BRM levels, we determined the BRM transcript and protein in single, double, and triple *brd* mutants. The BRM transcript changed moderately in the analyzed mutant lines (Figure S5). In contrast, BRM protein levels decreased significantly in the *brdx3* mutant (Figure 4b), in agreement with previous findings [11,19]. Notably, low levels of BRM protein were also detected in the *brd1/2* mutant, while they did not change much in *brd2/13, brd1/13*, and in each of the single mutants (Figure 4b). This indicates that BRD1 and BRD2 are critical to maintaining adequate levels of BRM protein and that they act in this process redundantly. The low level of BRM protein in *brd1/2* was unexpected, given that this mutant shows much weaker morphological changes than *brdx3* (Figure 4a). To confirm the observed changes in BRM protein levels, we generated *brm-1/*BRM-GFP lines in the double *brd* mutant backgrounds and estimated BRM-GFP levels using confocal microscopy and Western blotting. Consistently, BRM-GFP levels decreased significantly in the *brd1/2* background and less changed in *brd1/13* and *brd2/13* (Figure 4c–e). Therefore, the low level of BRM does not seem to be a direct cause of the observed mutant phenotypes.

### 2.5. BRD Subunits Are Necessary for the Integrity of the SWI/SNF Complex

As changes in BRM levels could not fully explain the different phenotypes of double and triple *brd* mutants, we speculated that they may result from the disturbance of the integrity of the complex. To find out whether this is the case, we performed BRM-GFP affinity purifications followed by mass spectrometry in the triple and double *brd* mutant backgrounds (Supplementary Data S6–S9). Consistent with the changed levels of BRM-GFP in *brd* mutants detected by Western blot and microscopy, BRM was identified in IP/MS experiments with a lower number of peptides in the *brdx3 brm-1*/BRM-GFP and *brd1/2 brm-1*/BRM-GFP lines compared with the control line *brm-1*/BRM-GFP, while the number of BRM-derived peptides decreased moderately or did not change in the *brd1/13* and *brd2/13* backgrounds, respectively (Figure S6). Importantly, the lack of BRD1/2/13 resulted in a total loss of identifications of several other SWI/SNF components, including SWI3C and SWP73B/A. In effect, ARP4 and 7 and BDH2 were the only non-catalytic subunits that could be detected in six IP/MS experiments in the *brdx3* background (Table 1). This result was unlikely to be caused by differences in the depth of MS analysis between different samples, since the total number of peptides detected in IPs performed in the control and *brdx3* mutant lines was on average similar and did not appear to correlate with the number of peptides derived from BRM (Figure S7). Together, these data indicate that the *brdx3* mutation strongly affects the ability of BRM to co-precipitate other complex subunits, showing that BRDs are crucial for maintaining not only the proper level of BRM protein, but also integrity of the entire SWI/SNF complex. Unlike *brdx3,* most subunits of the BRM complex could be identified in the double mutant backgrounds, showing that the presence of at least one BRD subunit is necessary for the formation of the complex (Table 1). Furthermore, among the immunoprecipitated subunits in IP of *brd1/2*, *brd1/13*, and *brd2/13*, the remaining BRD (BRD13, BRD2, BRD1, respectively) was present (Table 1), indicating that BRDs are incorporated into SWI/SNF independently, which is consistent with a previous report that BRDs interact directly with different core subunits, but not with each other [11].

**Figure 5:**
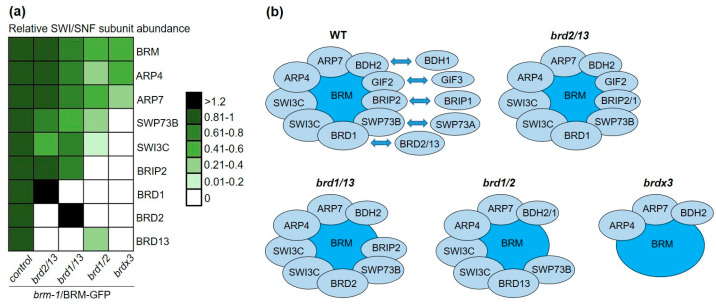
Mass spectrometry analysis of SWI/SNF complexes in *brd* mutant lines compared to the BRM-GFP control line: (**a**) relative abundance of the major subunits in complexes purified from the indicated Arabidopsis lines, normalized to BRM-GFP purifications of the control *brm-1*/BRM-GFP line; paralogs for which a small number of peptides were identified in the control line were not included in the calculations; (**b**) BRM complex assemblies detected in 2-week-old WT plants and *brd* mutants. The most abundant complex containing BRD1 remains in the *brd2/13* mutant, while other *brd* mutations lead to a decrease or loss of subunit identifications. The residual complex containing BRM, ARP, and BDH subunits is detected in the *brdx3* mutant.

Moreover, the lack of BRD proteins resulted in a decrease in the abundance of the detected subunits in the BRM-GFP IP (Figure 5a), likely reflecting loss of integrity and stability of all or a fraction of the complexes in the triple and in each of the double *brd* mutants, respectively. Comparing the double mutants, changes in subunit identification frequency and abundance were more pronounced in *brd1/2* than in *brd1/13* or *brd2/13* (Table 1, Figure 5a), suggesting that BRD1 and 2 contribute more than BRD13 to maintain a pool of stable BRM complexes. Furthermore, the relative abundances of BRD1 and BRD2 increased in the complexes isolated from the *brd2/13* and *brd1/13* mutant, respectively, while the abundance of BRD13 in the complex isolated from *brd1/2* remained similar to other subunits (Figure 5a). This further supports the notion that BRD13 is present in a lower number of SWI/SNF assemblies than BRD1/2 and suggests that BRD1 and BRD2 can replace each other but are not interchangeable with BRD13. Notably, the ability of BRM to form low abundant BRD13-containing complexes in the absence of BRD1/2 on the one side and severe decomposition of the BRM complex in the absence of BRD1/2/13 (Figure 5b) on the other provides a possible explanation for the observed phenotypic differences between *brd1/2* and *brdx3* mutants. For the latter, IP/MS data suggest that the amount of complete BRM assemblies is very low (below the detection limit of the method) or, more likely, only partial complexes composed of BRM, ARP4 and 7, and BDH2 exist in this mutant (Figure 5b).

### 2.6. BRD Subunits Are Required for the Assembly of the Complete BRM Complex

**Figure 6:**
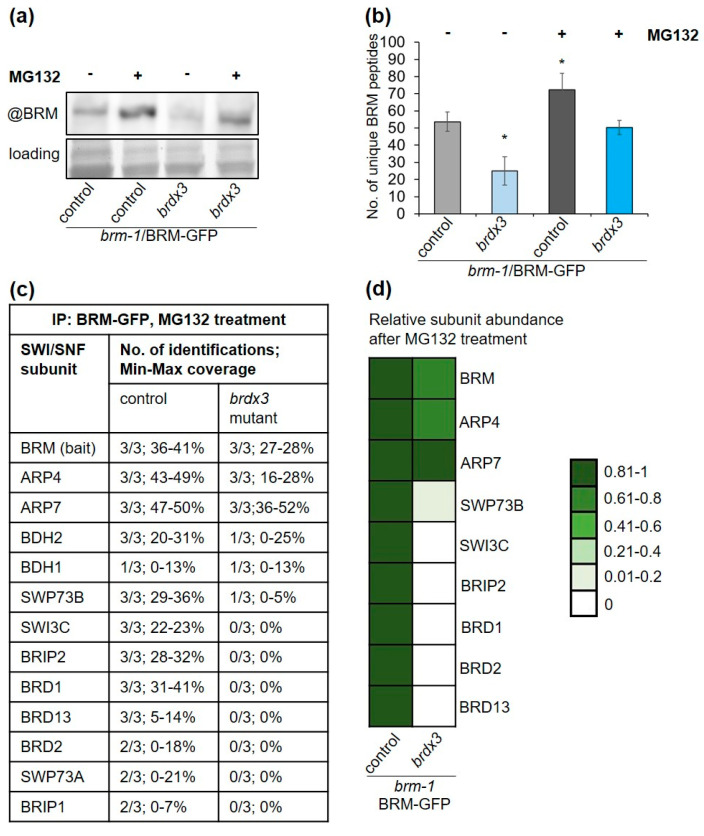
Assembly of the BRM complex is disturbed in the *brdx3* mutant: (**a**) immunoblot analysis of the BRM-GFP protein in *brm-1* (control) and *brm-1 brdx3* background with or without MG132 treatment; (**b**) number of BRM-derived peptides recovered by immunoprecipitation of BRM-GFP in the control and *brdx3* mutant with and without MG132 treatment; data presented as mean values ± s.d. of three biological replicates; asterisks indicate a significant difference from the untreated control line (Student’s *t* test, *p* < 0.01); (**c**) mass spectrometry analysis of SWI/SNF complexes isolated after treatment with MG132 from the control and *brdx3* mutant; number of identifications and the min–max coverage for each subunit are shown; (**d**) relative abundance of the major subunits in SWI/SNF complexes purified from the *brdx3* mutant compared to the control line after MG132 treatment; paralogs for which a small number of peptides were identified in the control line were not included in the calculations.

The identification of partial complexes in the *brdx3* mutant led us to speculate that BRM complexes in Arabidopsis may have a modular type of organization, with BRM-ARP-BDH forming one of the modules (catalytic module), similar to mammalian SWI/SNF complexes [27]. Moreover, BRDs may play a role in the assembly of this module with the rest of the complex. To test this hypothesis, we purified BRM complexes from *brm-1*/BRM-GFP and *brdx3 brm-1*/BRM-GFP lines after the plants were treated with proteasome inhibitor MG132. We reasoned that if the *brdx3* mutation affected only the stability of the BRM complex, then recovery of fully assembled complexes on MG132 should be observed. Treatment with MG132 had a positive effect on BRM-GFP protein levels assessed by Western blotting in *brm-1* and *brm-1 brdx3* lines (Figure 6a), in agreement with previous results [19,28].

**Figure 7:**
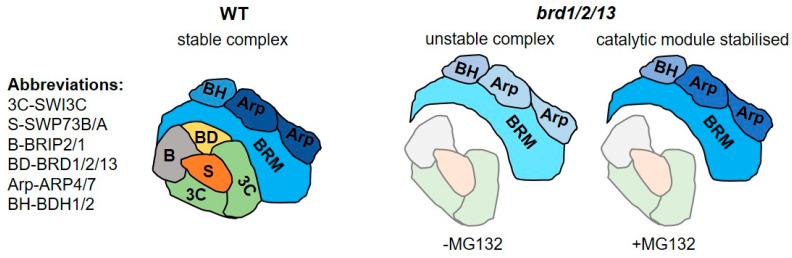
Schematic showing the role of the BRD subunits in the assembly of the complete BRM complex. Subunit abbreviations are indicated. Loss of all BRDs impedes the assembly of the complex and leads to a decrease in the stability of the catalytic module. In the presence of MG132, the catalytic module is stabilized in the *brdx3* mutant (dark blue), but the assembly remains inefficient.

The effect of MG132 was also evident in the IP/MS data, since the number of identified peptides corresponding to BRM was higher in treated than non-treated control plants (Figure 6b). The peptide counts of other major subunits also increased (Figure S8), indicating the stabilization of BRM complexes. Importantly, BRM peptide counts were also much higher in IP/MS data from treated *brdx3 brm-1/BRM-GFP*, reaching values comparable to those of the untreated control line (Figure 6b). Moreover, ARP4 and ARP7 were identified with higher peptide counts (Figure S8). However, identifications of other subunits were absent (SWI3C, BRIP2, BRIP1, SWP73A) or rare with very low coverage (SWP73B) under these conditions (Figure 6c,d and Figure S8), suggesting that full complexes could not be formed. In contrast, IP/MS after MG132 treatment in the double *brd* mutant backgrounds *brd1/2*, *brd1/13*, and *brd2/13* resulted in efficient recovery of full complexes containing one remaining BRD protein (Figure S9, Supplementary Data S10). In conclusion, in the absence of all BRD, only the catalytic module could be efficiently immunoprecipitated after inhibition of the proteasome, confirming the presence of residual complexes in the *brdx3* mutant and supporting the role of BRD1/2/13 in the assembly of the catalytic module with the rest of the complex (Figure 7).

## 3. Discussion

The aim of this work was to better understand the organization of SWI/SNF complexes formed by the BRM ATPase. The results of IP/MS experiments indicate that the BRM complex has a characteristic subunit composition consisting of ARP4 and 7, SWP73A/B, SWI3C, BRIP1/2, BRD1/2/13, GIF1/2/3, and BDH1/2 (Figure 1d,e and Supplementary Data S1,S2). The list of BRM-associated proteins obtained in our study is likely a complete set of subunits, as no other protein was reproducibly identified in eight BRM-GFP IP/MS experiments. Furthermore, IP/MS using BRD1-GFP resulted in the identification of almost the same set of proteins as in BRM-GFP purifications, showing that BRD1 represents a signature subunit of the BRM complex (Table S2). At the same time, a greater number of proteins homologous to SWI/SNF components, including both BRM-associated proteins and those not present in BRM-GFP purifications, could be immunoprecipitated using SWP73B-YFP and BDH2-GFP as bait (Table S2). This corroborates the specificity of the BRM-captured complexes and indicates that SWP73B and BDH2 are subunits of several different types of SWI/SNF assemblies, one of which is the BRM complex. In particular, BDH2-GFP captured a broad set of associated proteins comprising almost all characterized or predicted SWI/SNF subunits, including TPF1 and 2 proteins recently shown to be homologs of mammalian BAF45, as well as PSA1/2, BRD5, and OPF1/2 representing putative plant-specific subunits [20] (Table S2). Analyses of T-DNA mutants in the *BDH1* and *BDH2* genes revealed that BDH1/2 act redundantly to control developmental and physiological processes commonly affected by mutations in SWI/SNF subunits (Figure 2), supporting identification of BDH1/2 as components of the BRM and other SWI/SNF complexes. Furthermore, each of the bait proteins used in our experiments did not capture their paralogues, strongly suggesting that the SWI/SNF complexes use only one paralogue of the SWP73A/B, BDH1/2, GIF1/2/3, and BRD1/2/13 families. During the preparation of this manuscript, another report was published showing the composition of plant SWI/SNF complexes with similar conclusions regarding their composition, including the presence of BRD1/2/13 and BDH1/2 proteins as unique and non-unique subunits of the BRM complexes, respectively [29].

The limited diversity of subunits associated with Arabidopsis BRM can be regarded as unexpected, as two existing ATPase paralogs in mammals, BRM and BRG1, were shown to be part of different types of SWI/SNF complexes: BAF, PBAF, and non-canonical BAF (ncBAF) [26,27]. In contrast, the characteristic subunit composition of the BRM complex in Arabidopsis strikingly resembles the composition of only one of the SWI/SNF type, ncBAF [26,27]. In mammals, ncBAF is characterized by the presence of the GLTSCR and BRD9 proteins as unique signature subunits and the lack of BAF47 and ARID1/2, which are specific to the BAF and PBAF complexes. Similarly, the Arabidopsis BRM complex incorporates BRIP1/2 and BRD1/2/13 (homologs of GLTSCR and BRD9) and does not contain BSH and LFR, which represent subunits of BAF47 and ARID1/2-type, respectively (Figure 3). Furthermore, both the BRM complex and ncBAF contain a single variant of the SWI3 subunit. The finding that the BRM complexes in the vegetative stage contain SWI3C as the only representative of the four-member Arabidopsis SWI3 family is consistent with the highly similar morphological phenotypes of *swi3c* and *brm* null mutants [30]. The composition of the BRM complex is also consistent with published data on direct protein interactions. For example, the lack of BSH and LFR in BRM complexes is consistent with the findings that BSH and LFR can interact with SWI3A/B but not with SWI3C/D paralogs [7,25]. On the other hand, BRD1 interacts with SWI3C but not with SWI3A/B/D [11], consistent with both SWI3C and BRD1 representing unique subunits specific to BRM complexes.

Analyses of BRM complexes formed in the absence of two or three BRD uncovered the functions of these proteins in maintaining the entire complex. First, only low-abundance partial complexes could be purified using BRM-GFP expressed in the *brdx3* mutant, showing that BRDs are necessary for the integrity and stability of the complex. Second, BRM complexes were purified more efficiently from the *brd1/13* and *brd2/13* mutants than from *brd1/2* (Table 1; Figure 5a), indicating higher levels of assemblies containing BRD1 and BRD2 than BRD13 in the mutants. In particular, the BRD1-containing complexes isolated from the *brd2/13* mutant were highly abundant and similar to those purified from the control line, including the presence of subunits that are difficult to detect by mass spectrometry (BDH2, GIF2) (Table 1). Together with more frequent identifications of peptides corresponding to BRD1 than to BRD2 and BRD13 in the control line (Figure S8), this suggests that the complex containing BRD1 is the most common type of BRM assembly. Furthermore, the relative abundances of BRD1 and BRD2 increased in the purifications from *brd2/13* and *brd1/13* compared to the control line, suggesting that BRD1 and 2 can compensate for the loss of each other. This is not the case for BRD13, as its relative abundance in the complex isolated from *brd1/2* did not increase (Figure 5a). Thus, BRD1 appears to be the predominant BRD paralog present in BRM assemblies, and it can probably be exchanged by BRD2 but not by BRD13 within the same assembly. Third, while the *brd1/2* mutation results in a decrease in the BRM level that is comparable to that observed in *brdx3*, the *brd1/2* plant phenotype is much weaker than *brdx3* (Figure 4). This seeming discrepancy can be explained by the presence of BRM complexes containing BRD13 in the *brd1/2* mutant (Figure 5). Despite the low abundance, BRD13-containing complexes seem to be sufficient to ensure an almost normal development of *brd1/*2, in contrast to the residual complexes present in *brdx3*. Interestingly, although the *brdx3* mutant phenotype is relatively strong, it is less severe than the phenotype of null *brm-1* mutant [11,19], suggesting that the residual complexes are partially functional.

Finally, our findings provide insight into the architecture of BRM complexes. Studies in yeast and mammals revealed that SWI/SNF complexes have modular organization that probably reflects the assembly from distinct subcomplexes [27,31]. Notably, the composition of a partial complex identified in the *brdx3* mutant consisting of BRM, ARP4 and ARP7, and BDH2 (Figure 5), is reminiscent of the mammalian catalytic module composed of ATPase, β-actin and ACTL6A, and BCL7A/B/C [27]. This suggests that plant SWI/SNF complexes may have a modular type of organisation similar to mammalian complexes, showing functional conservation of subunit architecture. By inhibiting proteasome activity, we achieved an increase of BRM-GFP protein levels and stabilization of BRM complexes in the control line, as well as double *brd* mutants (Figure S9). However, in the *brdx3* mutant, MG-132 treatment did not allow the isolation of the entire complex. Instead, it resulted in stabilization of the catalytic module despite the fact that a relatively high level of BRM protein was reached (Figure 6b,c). Therefore, we propose that BRDs play an important role in the final step of assembly of the complete BRM complex. In the absence of BRD subunits (*brdx3* mutant), the assembly of all BRM complexes is inefficient, and the proteins that form the catalytic module can only co-purify with BRM (Figure 7). In addition, the inability to finalize the assembly probably leads to lower stability of the separate catalytic module. This would explain a decrease in the abundance of BRM and other components of the catalytic module, observed consistently in the *brdx3* as well as *brd1/2* and *brd1/13* mutants (Figure 4b–e, Figure 5a and Figure S8, Table 1). We note here that the requirement for the integrity of SWI/SNF complexes poses difficulties in studying other functions of BRD proteins. In particular, the *brdx3* mutation was shown to cause a significant decrease in BRM binding to its targets both at specific sites and genome-wide [11,19]. This effect can result from the loss of BRD-mediated targeting, the loss of complex integrity, or both, and further studies will be required to distinguish between these two effects.

## 4. Materials and Methods

### 4.1. Plant Lines

Wild-type *Arabidopsis thaliana* (WT) and all mutant lines were of the Columbia-0 (Col-0) ecotype. Mutant alleles *brm-1*, *brd1-2*, *brd1-5*, *brd2-1*, and *brd13-4*, as well as the BRM-GFP, *brm-1/BRM-GFP*, *brdx3 brm-1/BRM-GFP3*, *35S:GFP-AN3*, and SWP73B-YFP lines, have previously been characterized [4,8,11,13,32]. The *brd1/2 brm-1/BRM-GFP*, *brd1/13 brm-1/BRM-GFP*, and *brd2/13 brm-1/BRM-GFP* lines were constructed by crossing the *brm-1/BRM-GFP* plants with the respective *brd* double mutants. Insertion mutants in *BDH* genes were obtained from the Nottingham Arabidopsis Stock Centre. Three *BDH1* mutations and three *BDH2* mutations were initially tested: *bdh1-1* (SALK_053046), *bdh1-2* (SALK_152173), *bdh1-3* (SALK_046333), *bdh2-1* (SALK_060883), *bdh2-2* (SALK_042826), and *bdh2-3* (SALK_029285). The locations of the T-DNA insertions were confirmed by sequencing allele-specific PCR products and were as follows: 70 bp upstream of the translational start codon (ATG) (5′ UTR region) in *bdh1-1*, 86 bp downstream of ATG (1st exon) in *bdh1-2*, 256 bp downstream of ATG (1st intron) in *bdh1-3,* 103 bp upstream of ATG (promoter region) in *bdh2-1*, 277 bp upstream of ATG (promoter region) in *bdh2-2*, and 256 bp downstream of ATG (1st exon) in *bdh2-3.* Mutant alleles were selected for further analysis based on the position of the insertion and expression levels of the mutated genes. To generate the 35S:BRD1-GFP line, Arabidopsis Col-0 plants were transformed using the floral dip method using the *Agrobacterium tumefaciens* strain GV3101 containing pGWB605-BRD1 construct [11]. Finally, the 35S:BRD1-GFP line was crossed with the *brd1-5* mutant to generate the *brd1-5/35S:BRD1-GFP* line. To generate the line pBDH2: BDH2-GFP/bdh2, the genomic fragment encompassing *BDH2* was cloned into the pDONR201 vector, verified by sequencing, and transferred by LR reaction to the gateway-compatible vector pGWB604 [33]. The binary construct obtained was placed in *Agrobacterium tumefaciens* strain GV3101, which was then used to transform *bdh2-3* mutants using the floral dip method. Homozygous lines were identified by genotyping. The primers used for genotyping are listed in Table S3.

### 4.2. Growth Conditions

For all experiments, the seeds were surface sterilized with gaseous chlorine for 3–5 h and stratified for 2–3 d at 4 °C. Seeds were sown on a mixture of soil and vermiculite (3:1) or plated on half-strength MS medium containing 0.5% sucrose and 0.8% agar. The plants were grown under long day conditions (LD; 16 h light/8 h dark) at 22/19 °C. The flowering time was scored as the number of days after stratification (DAS). The number of rosette leaves and the days to flowering were counted after the main stem had bolted by 0.5 cm. For the germination assay, seeds were sown on MS plates supplemented with 1 or 10 µM PAC. To score the seed germination rate, plants were counted at 7 DAS. Seeds that did not show radicle emergence were scored as not germinated. For seedling growth (green cotyledon) assays, seeds were sown on MS plates supplemented with ABA at a concentration of 0.5 or 1 µM. Plants that had formed green cotyledons were counted at 7 DAS. For IP/MS analysis, seedlings were grown under LD conditions for 14 days at 22/19 °C in liquid ½ MS medium (0.5× Murashige and Skoog salts, 0.5% sucrose (*w*/*v*), 0.05% (*w*/*v*) MES, pH 5.7) supplemented with ½ MS vitamin solution (Sigma-Aldrich, Saint Louis, MO, USA), as described previously [11]. For proteasome inhibition, MG132 (Sigma-Aldrich) was added to the medium at 25 µM concentration 12 h before sample collection.

### 4.3. Protein Extraction and Immunoprecipitation

Immunoprecipitation experiments were performed according to the previously published protocol [11] with some modifications. Briefly, 4 g of ground plant material was resuspended in 12 mL of lysis buffer (50 mM Tris-HCl pH 7.5, 150 mM NaCl, 5 mM MgCl_2_, 1% Triton™X-100, 2% glycerol, 5 mM DTT, 1 mM PMSF) containing viscolase (A&A Biotechnology, Gdansk, Poland), protease inhibitors (Complete EDTA-free, Roche, Basel, Switzerland), and proteasome inhibitor MG132 (Sigma-Aldrich) and incubated 45–60 min. Cell lysates were cleared by filtering through two layers of Miracloth (Calbiochem, San Diego, CA, USA) and centrifuged at 20,000× *g* for 15 min at 4 °C. The cleared supernatant was subsequently mixed with 25 µL of GFP-Trap agarose beads (ChromoTek, Planegg, Germany). After 1 h at 4 °C, the beads were washed 2 times with lysis buffer and 5 times with washing buffer (50 mM Tris-HCl pH 7.5, 150 mM NaCl, 1% glycerol, 1 mM DTT). The immunoprecipitated proteins were then subjected to on-bead digestion. Agarose beads with bound proteins were suspended in 100 µL of 5 mM TCEP (Tris-(2-carboxyethyl) phosphine), 100 mM NH_4_HCO_3_, and incubated for 1 h at 60 °C. After that time, MMTS (S-Methyl methanethiosulfonate) was added to the final concentration of 8 mM. The samples were incubated for 10 min at room temperature. After that time 500 ng of trypsin was added and the samples were incubated overnight at 37 °C and centrifuged. The supernatants were collected and used for LC-MS analyses.

### 4.4. LC-MS/MS Analysis

LC-MS/MS analysis was performed with the use of Evosep One LC (Evosep Biosystems, Odense, Denmark) with Endurance EV-106 chromatographic column (15 cm × 150 µm, C-18 resin bead size 1.9 µm) installed, coupled with an Orbitrap Exploris 480 mass spectrometer (Thermo Fisher Scientific, Waltham, MA, USA) in the Laboratory of Mass Spectrometry, Institute of Biochemistry and Biophysics PAS (Warsaw, Poland). Peptides obtained by trypsin digestion were loaded onto disposable EvoTips by centrifugation and processed according to the manufacturer’s recommended protocol. EvoTips with bound peptides were placed in the Evosep One LC autosampler. The samples were analyzed after the manufacturer’s recommended 88 min method (gradient of acetonitrile with 0.1% formic acid (*v*/*v*) as the pairing agent). The mass spectrometer was operated in data-dependent mode working with a resolution of 60,000 for MS scans and 15,000 for MS/MS scans. A higher-collision energy (HCD) device was utilized for the fragmentation of peptides.

### 4.5. MS Data Analysis

The RAW data obtained were processed with Mascot Distiller (Matrix Science, London, UK). The resulting files were uploaded to the Mascot server installed in the Laboratory of Mass Spectrometry, IBB PAS and searched against the *A. thaliana* Tair 10 database (35,386 entries). Mascot search parameters used were: enzyme, trypsin; fixed modification used, carbamidomethyl (C); variable modifications, oxidation (M); peptide mass tolerance, 15 ppm; fragment ion mass tolerance, 0.01 Da; and up to two missed cleavages. Data were imported into MScan software version 2.0.5 (proteom.ibb.waw.pl), which allowed their filtration and presentation. We applied a false discovery rate (FDR) threshold of 0.05 for peptide identification and a Mascot score of at least 30 for protein identification. Only proteins with at least two matched high-confidence peptides were further processed. The experimental background proteins were subtracted based on the 8 control GFP IP/MS experiments processed using the same parameters. Data from IP/MS experiments are found in Supplementary Data S1–S11. Calculations of the mean number of peptides were performed for each genotype using data from the 3 biological replicates with the most total peptides corresponding to SWI/SNF subunits excluding bait.

### 4.6. Western Blot

The native BRM protein, as well as BRM-GFP, was detected by Western blotting as previously described using anti-BRM antibodies [34]. The samples analyzed were whole cell extracts from MS-grown seedlings and nuclear extracts from plants grown in soil. The relative BRM signal was quantified with ImageJ software version 1.53t (imagej.nih.gov/ij) through comparison with the protein level on the Coomassie-stained gel (loading control) or the unspecific signal present on the same membrane.

### 4.7. RNA Isolation and RT-qPCR

The expression of BRM was analyzed in aerial parts of 10-day-old seedlings grown on MS plates under LD conditions. RNA was extracted from plant material using a GeneJET RNA Purification Kit (Thermo Fisher Scientific) and then digested with TURBO™ DNase (Ambion). Total RNA (1 μg) was reverse transcribed using a Transcriptor First-Strand cDNA Synthesis Kit (Roche). RT-qPCR analyses were performed using SYBR Green I Master mix in a LightCycler 96 (Roche) with gene-specific primers. The level of the BRM transcript was normalized to that of the housekeeping gene *PP2a* [35]. Three biological replicates were examined for each genotype. The primers used for qPCR are listed in Table S3.

### 4.8. Localization Analysis

Full-length cDNAs corresponding to BDH1 and BDH2 were cloned into the vector pDONR201 and verified by sequencing. Corresponding entry clones were used in LR recombination reactions to transfer the cDNA fragments to the gateway-compatible expression vector pGWB605 [33]. The binary constructs obtained were used to transform *Agrobacterium tumefaciens* GV3101. Each pGWB605 construct was coexpressed in 6-week-old *Nicotiana benthamiana* leaves after leaf infiltration with GV3101 strains containing the tested construct plus an antisilencing agrobacterial strain expressing P19. In addition, the agrobacterial strain transformed with 35S::H2B-RFP was used to visualize the nuclei as described previously [11]. Fluorescence was analyzed 2 d after infiltration using a Nikon D-Eclipse C1 laser scanning confocal microscope.

### 4.9. Confocal Microscopy

Roots were stained with 10 μg/mL propidium iodide for 2 min, rinsed, mounted in water, and visualized after excitation with a 488-nm laser line. Fluorescence emission was collected from 590 to 700 nm (propidium iodide) and from 496 to 542 nm (GFP). The intensity of the fluorescence signal was analyzed with ImageJ software [36] and measured as an integrated density for at least 14 roots for each genotype. Confocal laser scanning microscopy was performed using a Nikon D-Eclipse C1 microscope.

## 5. Conclusions

Our studies have shown that the BRM-containing SWI/SNF complexes in Arabidopsis belong to the class of non-canonical BAF (ncBAF) complexes and include the subunits ARP4, ARP7, BDH2/1, GIF1/2/3, SWP73B/A, SWI3C, BRIP2/1, and BRD1/2/13. Analyses of complexes formed in *brd* mutants showed that BRD subunits are crucial for maintaining the integrity and stability of the complex and revealed the existence of a catalytic module, which appears to be assembled with other subunits in a BRD-dependent manner. Since the residual complexes detected in the *brdx3* mutant are likely partially functional, it remains to be determined to what extent they retain remodeling activity and how they are targeted at specific genomic loci. Further studies are also needed to fully understand the contribution of other subunits to the assembly of the complex.

## Figures and Tables

**Table 1:** Summary of BRM complex subunits identified in the *brd* mutant backgrounds compared with the control line BRM-GFP/*brm-1*. The numbers of identifications are shown.

SWI/SNF Subunit	BRM-GFP/*brm-1*	BRM-GFP/*brm-1* *brdx3*	BRM-GFP/*brm-1* *brd1/2*	BRM-GFP/*brm-1* *brd1/13*	BRM-GFP/*brm-1* *brd2/13*
BRM	8/8	6/6	4/4	4/4	3/3
ARP4	8/8	4/6	4/4	4/4	3/3
ARP7	8/8	6/6	3/4	4/4	3/3
SWP73B	8/8	0/6	3/4	4/4	3/3
SWI3C	8/8	0/6	3/4	4/4	3/3
BRIP2	8/8	0/6	0/4	4/4	3/3
BRD1	8/8	0/6	0/4	0/4	3/3
BRD2	4/8	0/6	0/4	4/4	0/3
BRD13	8/8	0/6	¾	0/4	0/3
SWP73A	2/8	0/6	0/4	0/4	0/3
BRIP1	2/8	0/6	0/4	0/4	2/3
BDH2	4/8	2/6	1/4	1/4	3/3
BDH1	1/8	0/6	1/4	0/4	0/3
GIF1	0/8	0/6	0/4	0/4	0/3
GIF2	1/8	0/6	0/4	0/4	1/3
GIF3	1/8	0/6	0/4	0/4	0/3

## Data Availability

All raw files related to mass spectrometry are deposited at proteome Xchange (http://www.proteomexchange.org/) under reference number 1-20211227-22140.

## Supplementary Materials

The supporting information can be downloaded at: https://www.mdpi.com/article/10.3390/ijms24043917/s1.
