# Supplementary material for: BRM Complex in Arabidopsis Adopts ncBAF-like Composition and Requires BRD Subunits for Assembly and Stability
Source: Int J Mol Sci. 2023 Feb 15;24(4):3917. doi: 10.3390/ijms24043917 (PMC9967331; doi:10.3390/ijms24043917)
Supplement: Supplementary file 1 [file ijms-24-03917-s001.zip › ijms-2216703-supplementary_Figures and Tables.pdf]

## SUPPLEMENTAL FIGURES AND TABLES

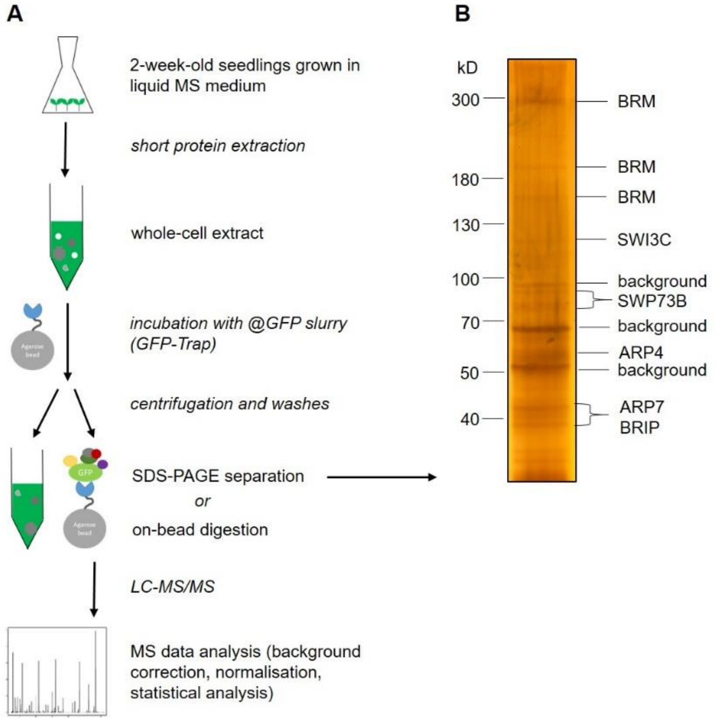


**Figure S1.** Isolation of SWI/SNF complexes from Arabidopsis seedlings. **(A)** Experimental setup of IP/MS analyses. (**B**) Silver-stained gel showing the proteins immunoprecipitated with BRM-GFP from the whole cell extracts of the *brm-1*/BRM-GFP line. Proteins were identified using LC-MS/MS.

**A**

```
sp|Q4VC05|BCL7A_HUMAN      MSGRSVRAETRSRAKDDIKRVMAAIEKVRKWEKKWVTVGDTSLR---------------- 44
sp|Q9BQE9|BCL7B_HUMAN      MSGRSVRAETRSRAKDDIKKVMAAIEKVRKWEKKWVTVGDTSLR---------------- 44
tr|Q9FLP2|At5g55210        MEGVGSRLSRTSSRYSGPAATAVFSGRVRKWKKKWVRVSTSSVGVFR----------ASK 50
tr|Q8GYJ0|At4g22320        MEGVGARLGRSSTRYGP---ATVFTGPVRKWKKKWVHVSPSSKKDNNNSSSGSAAAAASV 57
                           *.* . *    *   .    . .    ****:**** *. :*

sp|Q4VC05|BCL7A_HUMAN      -----------------IYKWVPVTEPKVDDKNKN-KKKGKDEKCGSEVTTP--ENSS-- 82
sp|Q9BQE9|BCL7B_HUMAN      -----------------IFKWVPVTDSKEKEKSKS-NSSAAREPNGFP--SD--ASAN-- 80
tr|Q9FLP2|At5g55210        SNGRNNSNNSNSPHHLLLHKWTPLTSATVTASDA----------NGSGETEESPKRRFRY 100
tr|Q8GYJ0|At4g22320        VNGGSNSDG-SNGSHLLLYKWAPLSQNGNGNEDGKSESNSPSEDTVATVAEDPPRRRFKY 116
                                            :.**.*::.     ..

sp|Q4VC05|BCL7A_HUMAN      -SPGMMDMHDDNSNQSSIADASPIKQENSSNSSPAPEPNSAVPSDG-TEAKVDEAQADGK 140
sp|Q9BQE9|BCL7B_HUMAN      -SSLLLEFQDENSNQSSVSDVYQLKVDSSTNSSPSPQQSESLSPAHTSDFRTDDSQP--- 136
tr|Q9FLP2|At5g55210        APIAMLEHREKV--ISKD---SEIEE---------------------------------- 121
tr|Q8GYJ0|At4g22320        VPIAVLEEQKKE--ITEIEEDDKIEE---------------------------------- 140
                               ::: :..    :.      ::

sp|Q4VC05|BCL7A_HUMAN      EHPGAEDASDEQNSQSSMEHSMNSSEKVDRQPSGD--SGLAAETSAI-SQDLEGVPPS-- 195
sp|Q9BQE9|BCL7B_HUMAN      --PTLGQEI-------LEEPSLPSSEVADEPPTLTKEEPVPLETQVVEEEEDSGAPPL-- 185
tr|Q9FLP2|At5g55210        -----TEEFD-------TESPLPKAVELDMN--LTDS----------------------- 144
tr|Q8GYJ0|At4g22320        -----DDKID-------EDNKVEQEDKVDEDKTVEESSEKKAEVEVEEKPDINDVPMEDI 188
                                 :           :  : .    *

sp|Q4VC05|BCL7A_HUMAN      --------------------------KKMKLEASQQNSEEM---------   210
sp|Q9BQE9|BCL7B_HUMAN      --------------------------KRFCVDQPTVPQTASES-------   202
tr|Q9FLP2|At5g55210        ----------DQ----TKEAKTGNLNLGLCLNSEGTEE------------   168
tr|Q8GYJ0|At4g22320        QQVEEKIVQDDEEKVVRQDLNESTVDLGLNLNANDADAENDPKEDKPLEE   238
                                                       : ::
```

**B**

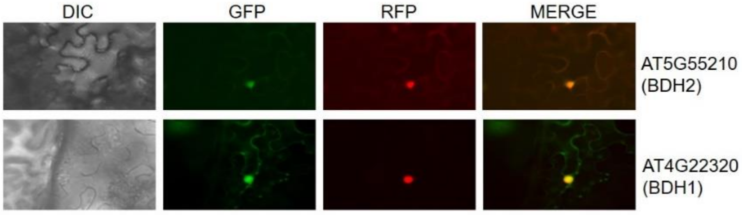


**Figure S2.** Analysis of the BCL7-like proteins AT4G22320 (BDH1) and AT5G55210 (BDH2). (**A**) Multiple sequence alignment of amino acid sequences of AT5G55210 and AT4G22320, as well as human BCL7A and BCL7B. Identical amino acids in all aligned sequences are marked with asterisks. (**B**) Nuclear localization of transiently expressed AT5G55210-GFP and AT4G22320-GFP fusions in tobacco epidermal cells examined by laser scanning confocal microscopy. Cells were co-transformed with H2B-RFP to visualize nuclei. DIC, differential interference contrast.

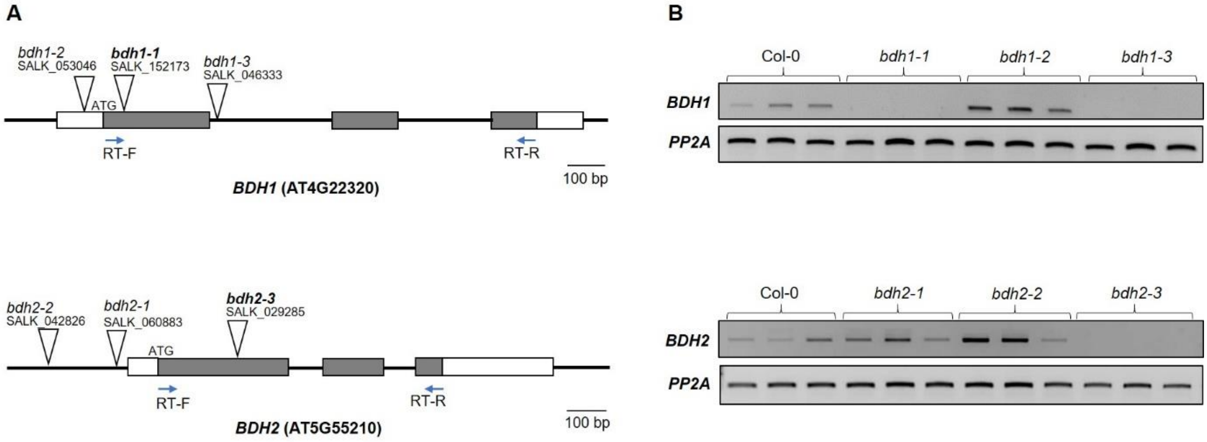


**Figure S3.** *BDH1* and *BDH2* mutant alleles. (**A**) Positions of T-DNA insertions in the *BDH1* and *BDH2* genes of mutant Arabidopsis lines. Arrows indicate positions of the primers used for RT-PCR. The mutant alleles used to generate double *bdh* mutants are bolded. (**B**) Full-length *BDH1* and *BDH2* transcripts detected by RT–PCR in wild-type and *bdh* mutants. Total RNA was isolated from 15-day-old seedlings grown under LD conditions, and the housekeeping gene *PP2A* was used as a control. Three biological replicates are shown for each line.

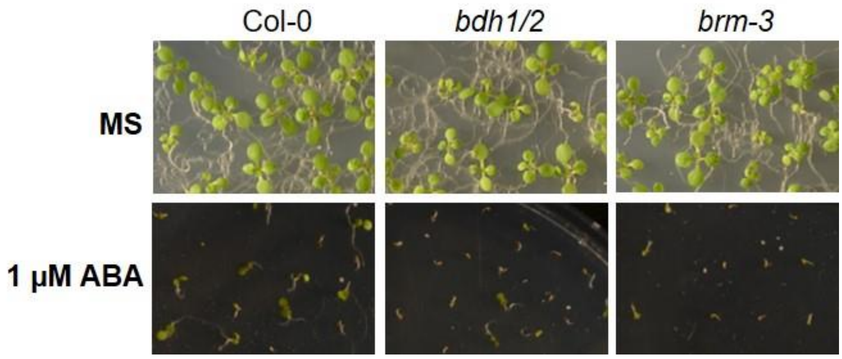


**Figure S4.** The *bdh1/2* double mutant shows hypersensitivity to ABA. Seedlings grown for x days on MS medium or in the presence of 1 µM ABA are shown. Bar = 10 mm.

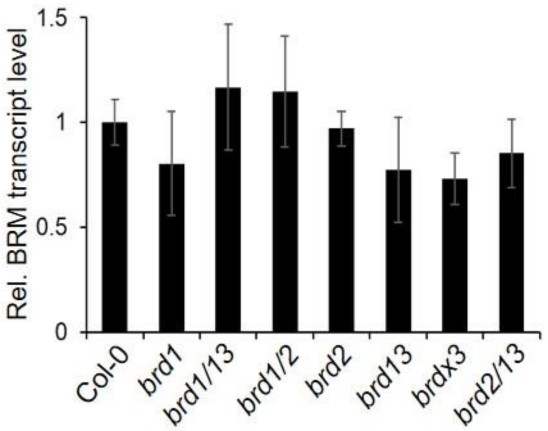


**Figure S5.** Quantitative RT-PCR analysis of BRM transcript levels in Col-0 wild-type and *brd* mutant lines. The *PP2A* housekeeping gene was used as normalization control. Means ± SD from 3 biological replicates are shown. The transcript level in Col-0 is set to 1.

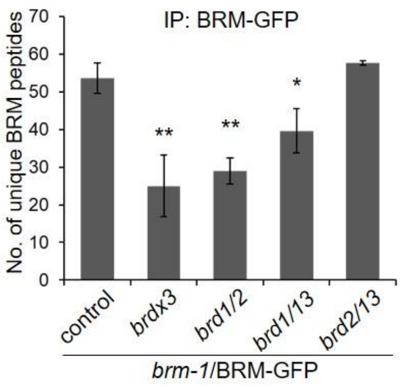


**Figure S6.** Mean number of BRM-derived peptides recovered by immunoprecipitation of BRM-GFP in the control and *brd* mutant lines. Means ± SD are shown. Asterisks indicate significant differences from the control line (Student's t test, *P < 0.05, **P < 0.01).

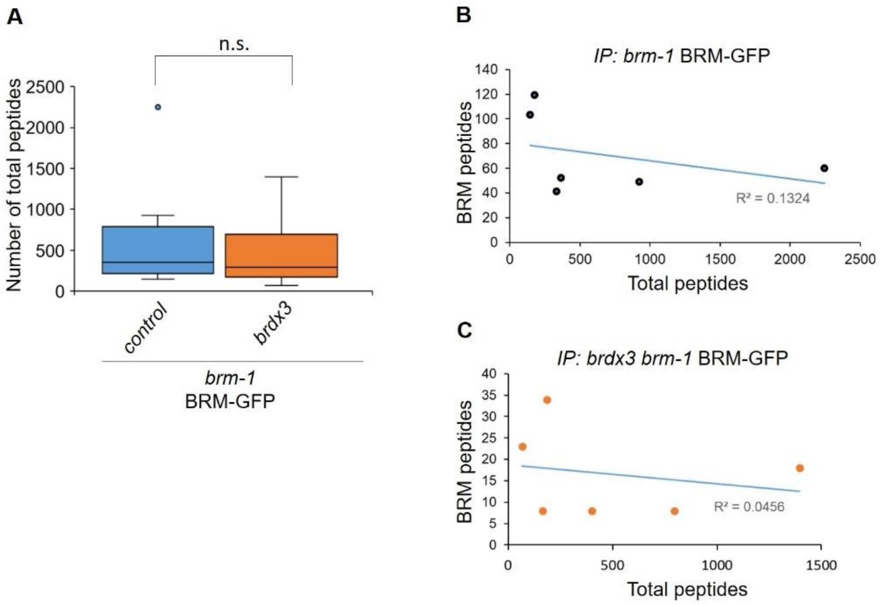


**Figure S7.** Comparison of total peptides and BRM-derived peptides detected in IP/MS experiments in the control line and *brdx3* mutant. (**A**) Mean number of total peptides. n.s., not significant (Student's t test). (**B, C**) The number of BRM-derived peptides does not correlate with the number of total peptides detected in the same experiments.

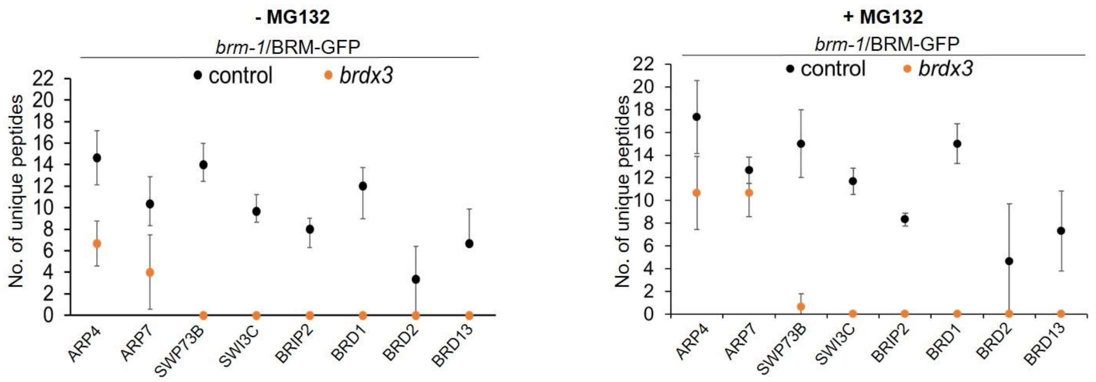


**Figure S8.** Peptides corresponding to the major subunits of the BRM complex identified in IP/MS experiments in the control and *brdx3* mutant**.** (**left**) Number of unique peptides recovered by immunoprecipitation of BRM-GFP in the control and *brdx3* mutant. (**right**) Number of unique peptides recovered by immunoprecipitation of BRM-GFP in the control and *brdx3* mutant after treatment with proteasome inhibitor MG132. Means ± SD are shown.

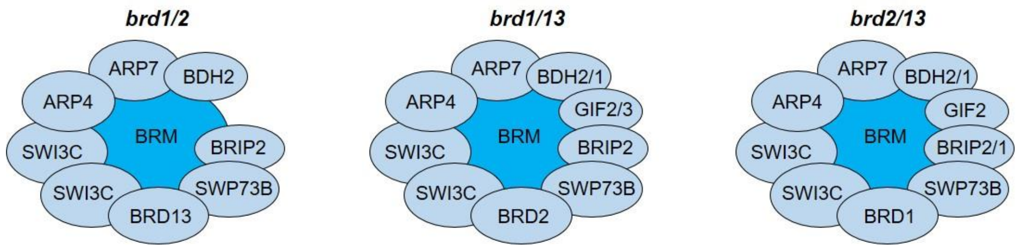


**Figure S9.** BRM complex assemblies purified from 2-week-old *brd* double mutants after treatment with MG132.

**Table S1** Defects of floral organs in *bdh* mutants compared to WT.

| | **WT** | ***bdh1*** | ***bdh2*** | ***bdh1/2*** |
|---|---|---|---|---|
| **Mean number of stamens** | 6±0 | 5.8 ± 0.4 | 5.7 ± 0.7 | 5.2 ± 0.4 |
| **Flowers with decreased number of stamens** | 0 (0%) | 5 (20%) | 9 (36%) | 19 (76%) |
| **Flowers with increased number of stamens** | 0 (0%) | 0 (0%) | 3 (12%) | 0 (0%) |
| **N** | 25 | 25 | 25 | 25 |

**Table S2** Identification of SWI/SNF subunits in IP/MS experiments using BRM-GFP, SWP73B-YFP, BDH2-GFP, or BRD1-GFP as baits. The numbers of identifications are shown.

| **Protein name** | **Gene** | **IP: BRM-GFP** | **IP: SWP73-YFP** | **IP: BDH2-GFP** | **IP: BRD1-GFP** |
|---|---|---|---|---|---|
| BRM | AT2G46020 | 8/8 | 2/2 | 2/2 | 2/2 |
| SWI3C | AT1G21700 | 8/8 | 2/2 | 2/2 | 2/2 |
| SWP73B | AT5G14170 | 8/8 | 2/2 | 2/2 | 2/2 |
| SWP73A | AT3G01890 | 2/8 | - | 2/2 | - |
| ARP7 | AT3G60830 | 8/8 | 2/2 | 2/2 | 2/2 |
| ARP4 | AT1G18450 | 8/8 | 2/2 | 2/2 | 2/2 |
| BRIP2 | AT5G17510 | 8/8 | 2/2 | 2/2 | 2/2 |
| BRIP1 | AT3G03460 | 2/8 | - | - | - |
| BRD1 | AT1G20670 | 8/8 | 2/2 | 2/2 | 2/2 |
| BRD2 | AT1G76380 | 4/8 | - | 2/2 | - |
| BRD13 | AT5G55040 | 8/8 | - | 2/2 | - |
| BDH1 | AT4G22320 | 1/8 | - | - | - |
| BDH2 | AT5G55210 | 4/8 | - | 2/2 | - |
| GIF1 | AT5G28640 | - | - | - | - |
| GIF2 | AT1G01160 | 1/8 | - | 1/2 | - |
| GIF3 | AT4G00850 | 1/8 | - | 1/2 | - |
| SYD | AT2G28290 | - | 2/2 | 2/2 | - |
| CHR12 | AT3G06010 | - | 1/2 | 2/2 | - |
| CHR23 | AT5G19310 | - | - | 2/2 | - |
| SWI3A | AT2G47620 | - | 1/2 | 2/2 | - |
| SWI3B | AT2G33610 | - | 1/2 | 2/2 | - |
| SWI3D | AT4G34430 | - | 2/2 | 2/2 | - |
| BSH | AT3G17590 | - | 1/2 | 2/2 | - |
| LFR | AT3G22990 | - | - | 2/2 | - |
| TPF1 | AT3G52100 | - | - | 2/2 | - |
| TPF2 | AT3G08020 | - | - | 1/2 | - |
| OPF1 | AT1G50620 | - | - | 2/2 | - |
| OPF2 | AT3G20280 | - | - | - | - |
| BRD5 | AT1G58025 | - | - | 2/2 | - |
| PSA1 | AT1G32730 | - | - | 2/2 | - |
| PSA2 | AT1G06500 | - | - | 1/2 | - |
| SHH2 | AT3G18380 | - | - | - | - |

**Table S3.** Oligonucleotides used in this study.

| Name | Sequence 5'-3' | Note |
|---|---|---|
| SALK_012963-F | ATCACCTCCTCAGGATCAAC | Genotyping of *brd1-2* mutant |
| SALK_012963-R | AAGGTGAAGACAACGACGAT | |
| GK219B04-02kz | TGAATTGTATTCCATTGCATTTGT | Genotyping of *brd1-5* mutant |
| GK219B04-03kz | TTGGAGTTGATGATGATGATCTGT | |
| SALK_025965-R | AATTAACGCGCCAAAATATC | Genotyping of *brd2-1* mutant |
| SALK_025965-F | CGCTGTTGGTTTCATTGTTTC | |
| SALK_208635C-F | GTCACGCTGATTCTGAGGAAG | Genotyping of *brd13-4* mutant |
| SALK_208635C-R | TGTATTGCATTGCATTTGAGC | |
| SALK_152173-F | CGACCGGAGAGATCTAAATCC | Genotyping of *bdh1-1* mutant |
| SALK_152173-R | ACTCCAAGGAATCAAAATGGC | |
| SALK_053046-F | CGACAATTAACGATTAGTTTCGG | Genotyping of *bdh1-2* mutant |
| SALK_053046-R | ACGACGAATCGTTTATCGTTG | |
| SALK_046333-F | TCCTTGGAGTAACTGGACTGG | Genotyping of *bdh1-3* mutant |
| SALK_046333-R | GGAGGTCAAAAAGTTCCCTTC | |
| SALK_060883-F | TCAACAGCAAGCATTACATGG | Genotyping of *bdh2-1* mutant |
| SALK_060883-R | TAACCGTCGCTGAGGTAAGTG | |
| SALK_042826-F | GCTGCGTTTCGACTAATCAAC | Genotyping of *bdh2-2* mutant |
| SALK_042826-R | CTCTGTACTTCCAGGGAAGCC | |
| SALK_029285-F | ACACAAGACATGTCCAAAGGC | Genotyping of *bdh2-3* mutant |
| SALK_029285-R | CAGTTTTTGCTTCCTGCAGAG | |
| Mut1L | GATTTCCCCAAATTCGATGC | Genotyping of *brm-1* mutant |
| Mut1R | GGGCTCTATGCAAATGCCTCA | |
| Lba1 | TGGTTCACGTAGTGGGCCATCG | Genotyping, T-DNA primer |
| Lbb1 | GCGTGGACCGCTTGCTGCAACT | Genotyping, T-DNA primer |

| | | |
|---|---|---|
| BDH1-RTF | GGTAGGTCCTCGACACGGTAC | RT-PCR |
| BDH1-RTR | GGCTTGTCCTCTTTCGGGTCG | |
| BDH2-RTF | ATGGAAGGTGTAGGATCGAG | RT-PCR |
| BDH2-RTR | TCATTCTTCAGTCCCTTCAG | |
| BDH1-qF | GTGCGACAAGATTTGAACGA | RT-PCR |
| BDH1-qR | CTAATGGCTTGTCCTCTTTCG | |
| BDH2-qF | TGGTAATTTGAATCTGGGACTGT | RT-PCR |
| BDH2-qR | GATCTAGAAGACGTCAAAGTGGA | |
| PP2A-F | TATCGGATGACGATTCTTCGTGCAG | RT-qPCR |
| PP2A-R | GCTTGGTCGACTATCGGAATGAGAG | |
| BRM-qF | TATCCTCCGGGTTCTGG | RT-qPCR |
| BRM-qR | CTCCGTTTCCGTTTTATCGT | |
| BDH1cds-Fattb | GGGACAAGTTTGTACAAAAAAGCAGGCT TCATGGAAGGAGTTGGTGCACG | Cloning of BDH1 cds |
| BDH1cds-Rattb | GGGACCACTTTGTACAAGAAAGCTGGGT CTTCTTCTAATGGCTTGTC | |
| BDH2cds-Fattb | GGGACAAGTTTGTACAAAAAAGCAGGCT TCATGGAAGGTGTAGGATCGAG | Cloning of BDH2 cds |
| BDH2cds-Rattb | GGGACCACTTTGTACAAGAAAGCTGGGT CTTCTTCAGTCCCTTCAG | |
